# Bruton tyrosine kinase inhibitor-related atrial fibrillation and its implications in the treatment of B-cell lymphoma

**DOI:** 10.3389/fcvm.2024.1408983

**Published:** 2024-07-26

**Authors:** Jun Du, Ze-Yu Chen, Xiao-Ran Gu, Ting Wang, Zou-Fang Huang

**Affiliations:** ^1^ Department of Hematology, Renji Hospital, School of Medicine, Shanghai Jiao Tong University, Shanghai, China; ^2^ Shanghai Jiao Tong University School of Medicine, Shanghai, China; ^3^ Ganzhou Key Laboratory of Hematology, Department of Hematology, The First Affiliated Hospital of Gannan Medical University, Ganzhou, Jiangxi, China

**Keywords:** atrial fibrillation, Bruton tyrosine kinase inhibitors, cardiovascular toxicity, lymphoma, Ibrutinib

## Abstract

Adverse events of atrial fibrillation (AF) have been commonly reported in lymphoma patients in treating Bruton's tyrosine kinase inhibitors (BTKi). The incidence rate of AF can vary depending on the specific types of BTKi and the patient population. Totally 45 published studies have revealed that the overall incidence rate of AF is 5% (95% CI 4%–7%). By performing a subtype single-rate analysis, the second-generation BTKi shows a lower AF incidence rate and lower cardiovascular toxicity. In the subtype single-rate analysis, we conclude the different AF incidence rates of Ibrutinib (10%, 95% CI 7%–13%), Acalabrutinib (4%, 95% CI 1%–6%), Orelabrutinib (0%, 95% CI 0%–1%), and Zanubrutinib (0%, 95% CI 0%–1%). The comprehensive analysis of AF inspires us to better predict and manage AF and other cardiovascular events in treating lymphoma. Meticulous evaluation, collaboration between cardiologists and hematologists, and discovery of new biomarkers are essential for its management.

## Introduction

### The applications of BTKi in the treatment of lymphoma

BTK inhibitors are a kind of small molecule targeting the critical component BTK on the signal pathway of B Cells, related to B cell proliferation and survival, making it a significant therapeutic target for B-cell lymphoma (1, 2). BTK inhibitors have gained considerable attention in recent years due to their demonstrated efficacy in treating B-cell lymphoma (3) (Table 1).

**Table 1:** The overall introduction of different BTK inhibitors.

	First-generation	Second-generation			Third-generation
	Ibrutinib	Acalabrutinib	Zanubrutinib	Orelabrutinib	Pirtobrunib
Company	AbbVie/Johnson	AstraZeneca	BeiGene	InnoCare Pharma	Eli Lilly
First approved	2013.11 (NDA 205552)	2017.10 (NDA 210259)	2019.11 (NDA 213217)	2020.12 (H20200016)	2023.1 (NDA 216059)

### Indications and adverse events of BTK inhibitors

Since the emergence of the first-generation BTKi, Ibutinib, it has become a critical targeted drug for treating lymphoma, showing a good prognosis and relatively few side effects. The BTKi have been approved for marketing in many countries, and have been listed in chronic lymphocytic leukemia or small lymphocytic lymphoma (CLL/SLL) (4), mantle cell lymphoma (MCL) (5), Waldenstrom's Macroglobulinemia (WM) (6) and marginal zone lymphoma (MZL) (7), etc. Also, the different doses of BTKi are being tested, and indications' ranges are expanding to other B cell lymphoma or immune diseases. The different combinations of BTKi and other medications are being explored, like rituximab, Obinutuzumab, and venetoclax, for better outcomes and fewer side effects.

We may discover that the indications of BTKi are gradually expanding. Some of them have been proven while more indications are still in clinical trials. More than relatively common types of lymphoma, BTKi are in trials with central nervous system lymphoma (CNSL, NCT04438044) (8), large B cell lymphoma (DLBCL, NCT01855750) (9), and follicular lymphoma (FL, NCT02343120) (10). Furthermore, BTKi also plays a more curial role in autoimmune diseases, including multiple sclerosis (MS, NCT02975349) (11), systemic lupus erythematosus (SLE, NCT02975336) (3), and rheumatoid arthritis (RA, NCT03233230) (12).

In conclusion, we may find the importance and high risk of the incidence of AF in treating B-cell lymphoma. In contrast, the reports of AF and other cardiovascular events vary from each other. Thus, we may conclude different reports and have a more detailed description of AF in the different types of BTKi. Furthermore, we may find a better way to manage BTKi-related AF, improving the prognosis of lymphoma patients.

## Methods

**Figure 1:**
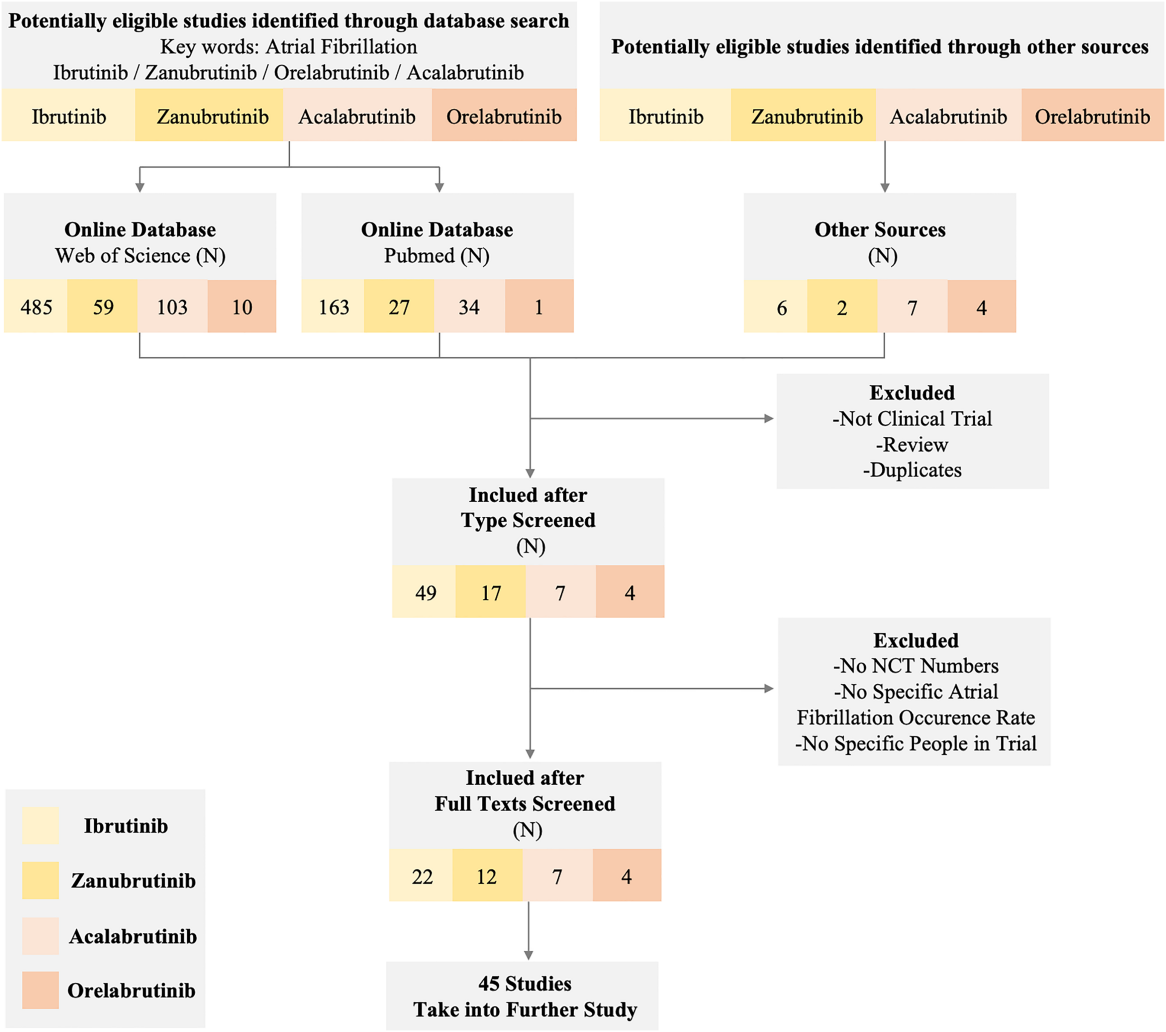
Flow diagram of the study. The diagram of the study shows the inclusion and exclusion criteria and a specific number of studies we obtained from different sources. We set atrial fibrillation and corresponding BTK inhibitors as keywords to exclude non-clinical trial studies and reviews. Eventually, 45 studies in different BTK inhibitors were taken into further research.

Due to the wide use of the BTKi in treating lymphoma and the noteworthy AEs of cardiotoxicity like AF in the clinical trial or normal therapeutic use, we collected related reports and paid special attention to AF events. Through extensive reading, different previous clinical trials and research suggest that diverse BTKi may act differently in the safety of AF. To further compare the safety of different generations of BTKi and have more reassuring guidance on clinical treatment, we systematically collected the clinical trials paper with NCT number (except Orelabrutinib) mentioned AF incidence rate. The data are collected from the most commonly used databases like Pubmed and Web of Science with keywords like BTKi and AF. All the clinical trials are collected from the published papers from 2013 to 2022, among a total of 657 papers from Web of Science and 225 papers from PubMed using the same strategy. After type filtering, eliminating duplicates, and screening contents, we finally reduced the scope to 45 papers Figure 1.

After overall criteria and careful reading, we finally collected papers on Ibrutinib (*N* = 22), Zanubrutinib (*N* = 12), Acalabrutinib (*N* = 7), and Orelabrutinib (*N* = 4). We mainly collect different AF incidence rates, related AE incidence rates, sample size, median age, severity, remark, etc.

## Results

### The AF incidence with BTK inhibitors

Through a comprehensive single-rate analysis encompassing data extracted from forty-two clinical trials conducted within the period spanning 2013–2022, we have concluded that the overall incidence rate of AF is relatively accurate at 5% (95% CI 4%–7% in the random effects model). It has been observed that second-generation BTKi exhibits fewer serious adverse events. Notably, the occurrence rate of AF displays significant variability, spanning from 0% to 35%. Having established an overall incidence rate of 5%, comparing the different BTKi therapies could provide valuable guidance for clinical treatment, particularly for patients with high cardiovascular risks. The heterogeneity observed between the four types of BTKi is evident (*I*^2 ^= 85% > 50%, *p* < 0.01), indicating the need for subtype analysis to uncover differences and explore the underlying causes of heterogeneity. Through subtype analysis, we found the AF incidence rates for Ibrutinib (10%, 95% CI 7%–13% in random effects model) (Table 2), Acalabrutinib (4%, 95% CI 1%–6% in random effects model) (Table 3), Orelabrutinib (0%, 95% CI 0%–1% in common effects model) (Table 4), and Zanubrutinib (0%, 95% CI 0%–1% in common effects model) (Table 5).

**Table 2:** Incidence of atrial fibrillation or flutter in ibrutinib.

Author, year of publication	Study design	Population	Sample size	Median age (y)	Median follow-up months	Atrial fibrillation or flutter	Severity	Cardiac-related AEs	Remark	NCT Number
Byrd (13)	RCT Phase III	R/R CCL/SSL	195	67y (range, 30y–86y)	9.4 months (range, 0.1–16.6)	10/195 (5.1%)	N/A	N/A	AF in the Ibrutinib group of 10 patients is higher than the one patient in the ofatumumab group.	NCT 01578707
Burger (14)	Phase III	Frontline CCL/SSL	136	73y	18.4 months	8/136 (5.9%)	6/8 (75.0%) Grade 2 2/8 (25.0%) Grade 3	6/135 (4.4%) Hypertension	Most AF patients have a history of cardiac events. There is a significantly higher ibrutinib than chlorambucil.	NCT 01722487
Farooqui (15)	Phase II	Frontline R/R CLL/SLL	86	(35/86) > 65y (51/86) > 18y	28 months	14/86 (5.6%)	11/14 (78.6%) Grade 2 3/14 (21.4%) Grade 3	N/A	The research with a higher AF incidence rate may owe it to longer follow-up time.	NCT 01500733
Teron (6)	Prospective study	WM	63	63y (range, 44y–86y)	N/A	3/63 (4.8%)	2/3 (66.7%) Grade 2 1/3 (33.3%) Grade 3	1/63 (1.6%) Sinus tachycardia Grade 2	The AF patients with *MYD88^L265P^*or *CXCR4^WT^*are all with AF history, and their overall toxic effects are moderate.	NCT 01614821
Chanan-Khan (16)	PC DB Phase III	CLL/SLL	289	64y (range, 31y–86y)	17 months (range, 13.7–20.7)	21/289 (7.3%)	N/A	89/289 (31%) Bleeding	Overall, a low incidence of AF was seen in BTKi therapy and chemoimmunotherapy.	NCT 01611090
Wang (17)	SC Phase II	R/R MCL	50	67y (range, 45y–86y)	16.5 months (range, 12.1–19.3)	7/50 (14.0%)	1/7 (14.3%) Grade 1–2 6/7 (85.7%) Grade 3	14/50 (28.0%) Hypertension	The occurrence of AF mainly owes to age and exposure to cardiac toxicity.	NCT 01880567
Ahn (18)	Phase II	CLL	51	66y (range, 33y–85y)	57.6 months	18/86 (20.9%)	13/18 (72.2%) Grade1–2 5/18 (27.8%) Grade 3	N/A	The therapy with Ibrutinib for more than five years is similar to earlier reports. Patients with TP53 aberration also reach durable responses.	NCT 01500733
Dimopoulos (19)	Phase III	WM	75	70y (range, 36y–89y)	26.5 months	11/75 (14.7%)	2/11 (18.2%) Grade1–2 9/11 (81.8%) Grade ≥3	10/75 (13.3%) Grade ≥3 Hypertension 38/75 (50.7%) Bleeding	A higher incidence of AF is observed in the Ibrutinib-Rituximab group compared to placebo-rituximab after 26 months.	NCT 02165397
Treon (20)	N/R	TN WM	30	67y (range, 43y–83y)	14.6 months	3/30 (10.0%)	3/3 (100.0%) Grade 2	4/30 (13.3%) Hypertension	Ibrutinib is well tolerated overall in TN patients with WM and has no unexpected toxicities. Patients with AF can usually be managed with medication without dose reduction.	NCT 02604511
Davids (21)	MC SA Phase II	TN CLL	85	55y (range, 50y–58y)	16.5months (range, 10.6–34.1)	5/85 (5.9%)	2/5 (40.0%) Grade1–2 3/5 (60.0%) Grade 3	6/85 (7.1%) Hypertension	The ibrutinib plus FCR (Fludarabine, Cyclophosphamide, and Rituximab) regimen has relatively few grades ≥ 3 AEs and few lead to discontinuations.	NCT 02251548
Jain (22)	Phase II	TN CLL	80	65y (range, 26y–83y)	14.8 months	12/80 (15.0%)	4/12 (33.3%) Grade 1–2 8/12 (66.7%) Grade 3–4	11/80 (13.8%) Hypertension	The combination therapy of Ibrutinib and venetoclax is effective and tolerable. The safety profile is similar to the monotherapy of Ibrutinib or venetoclax.	NCT 02756897
Munir (23)	MC Phase III	R/R CLL/SLL	195	N/A	41 months (range, 0.2–71.1)	24/195 (12.0%)	N/A	41/195 (21%) Hypertension 19/195 (10%) Bleeding ≥ Grade 3 2/195 (1.0%) Ventricular tachyarrhythmia 9/195 (4.6%) Heart failure	The overall prevalence of AF is similar to the earlier report. Most AEs, except hypertension, gradually decrease with treatment.	NCT 01578707
Nastoupil (24)	MC Phase I	CLL/SLL/ B-NHL	46	62y (range, 56y–67y)	15 months (range, 6.8–24.5)	2/46 (4.3%)	N/A	6/46 (13.0%) Hypertension	The study shows the tolerable safety profile of a triplet regimen (Ibrutinib, ublituximab, and umbralisib) with only one patient leading to discontinuation.	NCT 02006485
Tam (25)	Phase III	WM	98	(29/99) ≤ 65y (70/99) > 65y	19.4 months	15/98 (15.3%)	11/15 (73.4%) Grade 1–2 4/15 (26.6%) Grade ≥3	16/98 (16.3%) Hypertension	AF and hypertension are reported in higher frequency in the Ibrutinib group compared with Zanubrutinib.	NCT 03053440
Byrd (26)	Phase III	CLL	263	65y (range, 28y–88y)	40.9 months (range, 0.0–59.1)	41/263 (15.6%)	32/41 (78.0%) Grade 1–2 9/41 (22.0%) Grade ≥3	60/263 (22.8%) Hypertension 5/263 (1.9%) Ventricular arrhythmia or cardiac arrest	Compared with Acalabrutinib, more AEs like AF or hypertension are observed, and a five times higher rate of discontinuation due to cardiac events in the Ibrutinib group.	NCT 02477696
Sharman (27)	MC	R/R CLL	126	66y/67y (range, 62–74)	41.6 months (range, 36.7–47.3)	5/126 (4.0%)	N/A	N/A	The rate of incidence of AF in the Ublituximab plus ibrutinib group (four, 7%) is higher than Ibrutinib alone group (one, 2%).	NCT 02301156
Treon (28)	N/R	R/R WM	63	63y (range, 44y–86y)	59 months	6/63 (9.5%)	5/6 (83.3%) Grade 2 1/6 (16.7%) Grade 3	4/63 (6.3%) Hypertension 1/63 (1.5%) Hypotension	Ibrutinib responses are affected by *MYD88* and *CXCR4* mutation but with no unexpected AEs for its tolerable profile.	NCT 01614821
Trotman (29)	Phase III	WM	31	67y (range, 47–90)	58 months (range, 9–61)	0/31 (0.0%)	N/A	3/31 (9.7%) Grade ≥ 3 Hypertension	Ibrutinib is tolerated with no AF events, primarily low-grade AEs, and without the case of discontinuation of the treatment.	NCT 02165397
Castillo (30)	SG Phase II	TN WM	90	67y (range, 43y–83y)	50 months	6/30 (20.0%)	6/6 (100.0%) Grade 2	5/30 (16.7%) Hypertension 1/30 (3.3%) Cardiac arrest	All patients with AF can be managed medically and continue treatment, and not seen as a contraindication for Ibrutinib.	NCT 02604511
Jain (31)	SA Phase II	MCL	50	71y (range, 69y–76y years	45 months (range, 24–56)	17/50 (34.0%)	1/17 (5.9%) Grade 1 5/17 (29.4%) Grade 2 11/17 (64.7%) Grade ≥ 3	9/50 (18.0%) Bleeding 12/50 (24.0%) Hypertension	Patients who develop AF have higher baseline cardiac risk factors and ECG abnormalities than non-AF patients.	NCT 01880567
Langerbeins (32)	Phase III	TN CCL	158	64y (range, 38y–85y)	31 months	19/158 (12.0%)	9/19 (47.4%) Grade 1–2 10/19 (52.6%) Grade ≥3	16/158 (10.1%) Hypertension	Compared with the placebo, the occurrence of AE is similar, while Ibrutinib is associated with more cardiovascular and bleeding events.	NCT 02863718
Wang (33)	SC, SA Phase II	Frontline MCL	131	56y (range, 49y–60y)	42 months (range, 30–54)	5/131 (3.8%)	3/5 (60.0%) Grade 1 1/5 (20.0%) Grade 2 1/5 (20.0%) Grade 3	20/131 (15.2%) Hypertension	There are few significant AEs in the Ibrutinib–rituximab group.	NCT 02427620

RCT, randomized clinical trial; SG, sequential group; SC, single-center; MC, multiple-center; SA, single-arm; PC, placebo-controlled; DB, double-blind; CLL, Chronic Lymphocytic Leukemia; SLL, small lymphocytic lymphoma; MCL, mantle-cell Lymphoma; FL, follicular lymphoma; WM, Waldenstrom's macroglobulinemia; CNSL, central nervous system lymphoma; MZL, marginal zone lymphoma; R/R, Relapsed/refractory disease; TN, treatment naive; N/A, not acquired; AF, artificial fibrillation; AEs, adverse events; BTKi, Bruton's tyrosine kinase inhibitor.

**Table 3:** Incidence of atrial fibrillation or flutter in acalabrutinib.

Author, year of publication	Study design	Population	Sample size	Median age (y)	Median follow-up months	Atrial fibrillation or flutter	Severity	Cardiac-related AEs	Remark	NCT Number
Awan (34)	Phase 1/2	CLL/SLL	33	64y (range, 50y–82y)	9.5 months (range, 0.5 to 20.6)	2/33 (6.0%)	1/2 (50.0%) Grade 2 1/2 (50.0%) Grade 3	N/A	Acalabrutinib is usually tolerable and effective for patients with ibrutinib intolerance.	NCT 02029443
Wang (35)	MC, SA Phase II	R/R MCL	124	68y (range, 61y–75y)	15.2 months	0/124 (0.0%)	N/A	N/A	AF was not observed,while longer follow-up and other randomized studies are needed to further explore.	NCT 02213926
Byrd (36)	Phase 1b/2	R/R CLL/SSL	134	66y (range, 42y–85y)	41.0 months	10/134 (7.5%)	6/10 (60.0%) Grade1–2 4/10 (40.0%) Grade ≥3	N/A	The report supports the previous findings and provides added proof of endurance and tolerability, and the 100 mg twice-daily dose may be more effective.	NCT 02029443
Sharman (37)	Phase III	TN CLL	179	70y(range, 66y–75y)	28.3 months	7/179 (3.9%)	N/A	4/179 (2.2%) Hypertension Grade ≥3	Compared with chemoimmunotherapy, Acalabrutinib with Obinutuzumab or not has a more effective outcome and higher incidence rate of AF (7/179 vs 1/169).	NCT 02475681
Ghia (38)	MC Phase III	R/R CLL	154	68y(range, 32y–89y)	16.1 months (range, 0.03–22.4)	3/154 (1.9%)	N/A	2/195 (1.0%) Ventricular tachyarrhythmia 5/154 (3.2%) Hypertension 9/195 (4.6%) Heart failure	Acalabrutinib-related AEs are similar to the previous studies. Few serious AEs and few AEs leading to discontinuation.	NCT 02970318
Byrd (26)	MC Phase III	CLL	266	65y(range, 28y–88y)	40.9 months (range, 0.0–59.1)	24/266 (9.0%)	12/24 (50.0%) Grade ≥3	23/266 (8.6%) Hypertension	The first randomized phase III Acalabrutinib versus Ibrutinib trail shows a five-fold higher discontinuation rate because of cardiac events, 2.4 times higher AF rate, and shorter median onset time in ibrutinib.	NCT 02477696
Strati (39)	MC Phase II	R/R sMZL	43	69y (range, 42y–84y)	13.3 months (range 0.5–45.5)	0/43 (0.0%)	N/A	2/43 (4.7%) Hypertension	The lower incidence of AF with Acalabrutinib might be interpreted with caution due to the shorter follow-up.	NCT 02180711

MC, multiple-center; SA, single-arm; CLL, chronic lymphocytic leukemia; SLL, small lymphocytic lymphoma; R/R, relapsed/refractory disease; TN, treatment naive; N/A, not acquired; AF, artificial fibrillation; AEs, adverse events; BTKi, Bruton's tyrosine kinase inhibitor.

**Table 4:** Incidence of atrial or flutter in orelabrutinib.

Author, year of publication	Study design	Population	Sample size	Median age (y)	Median follow-up months	Atrial fibrillation or flutter	Severity	Cardiac-related AEs	Remark	NCT Number
Xu (40)	MC Phase II	R/R CLL/SLL	80	N/A	6.3 months (range, 0.4–13.7)	0/80 (0%)	N/A	No significant AEs	The improvement in selectivity allows Orelabrutinib to have an improvement in selectivity and no report of significant AEs.	N/A
Song (41)	MC Phase II	R/R MCL	106	N/A	15.0 months	0/106 (0%) ≥Grade 3	N/A	N/A	Orelabrutinib is safe and well tolerated, with a better selection for BTKi therapy.	N/A
Zhou (42)	SA MC Phase II	R/R WM	47	63y (range, 56y–68y)	10.5 months	0/47 (0%) ≥Grade 3	N/A	N/A	Orelabrutinib shows favorable safety and tolerability with fewer AEs and promising treatment efficacy in R/R WM patients.	N/A
Wu (8)	SG	CNSL	23	55.0y (range,41.2y -68.8y)	4.5 months (range, 2.9–5.8)	0/23 (0%)	N/A	N/A	It is the first study for patients with CNSL and shows a favorable outcome and mild, tolerable, and controllable AEs.	N/A

SG, sequential group; MC, multiple-center; SA, single-arm; cll, chronic lymphocytic leukemia; SLL, small lymphocytic lymphoma; MCL, mantle-cell lymphoma; WM, Waldenstrom's macroglobulinemia; CNSL, Central nervous system lymphoma; R/R, relapsed/refractory disease; N/A, not acquired; AF, artificial fibrillation; AEs, adverse events; BTKi, Bruton's tyrosine kinase inhibitor.

**Table 5:** Incidence of atrial fibrillation or flutter in zanubrutinib.

Author, year of publication	Study design	Population	Sample size	Median age (y)	Median follow-up months	Atrial fibrillation or flutter	Severity	Cardiac-related AEs	Remark	NCT Number
Tam (43)	MC Phase I	R/R CLL/SLL	144	Part 2 69y (range, 24y–87y)	Part 2 13.7 months (range, 0.4–30.5)	1/144 (0.7%)	1/1 (100%) Grade 2	N/A	The limited incidence rate of AF in Zanubrutinib may not increase risks though the mechanism of toxicity is not yet precise.	NCT 02343120
Dimopoulos (44)	Phase III	WM	28	72y (range, 39y–87y)	17.9 months	1/28 (3.6%)	1/1 (100%) Grade 1	3/28 (10.7%) Hypertension	Zanubrutinib generally has low cardiotoxicity in AF and other related side effects.	NCT 03053440
Song (45)	SA Phase II	R/R MCL	86	60.5y	18.4 months	0/86 (0.0%)	N/A	13/86 (15.1%) Hypertension	The safety of Zanubtutinib is more effective in prolonging treatment or reaching better outcomes due to higher selectivity.	NCT 03206970
Tam (46)	MC Phase 1b	FL CLL/SLL	81	N/A	N/A	0/81 (0.0%)	N/A	7/81 (8.6%) Hypertension	Compared with other BTKi, there is no AF case and a lower incidence rate of severe AEs in Zanubrutinib.	NCT 02569476
Tam (25)	Phase III	WM	101	(41/102) ≤ 65y (64/102) > 65y	19.4 months	2/101 (2.0%)	2/2 (100%) Grade 1–2	11/101 (10.9%) Hypertension	Though AF is a common complication in BTKi therapies, Zanubrutinib shows a lower AF incidence and a tolerable profile due to higher selectivity.	NCT 03053440
Trotman (47)	MC Phase 1/2	WM	77	67y (range, 40y–87y)	R/R 36.0 months TN 23.5 months	4/77 (5.2%)	1/4 (25.0%) Grade 1 2/4 (50.0%) Grade 2 1/4 (25.0%) Grade 3	12/77 (15.6%) Hypertension	No patients require dose reductions or treatment discontinuation for the long-term tolerable treatment.	NCT 02343120
Xu (48)	SG Phase II	R/R CLL/SLL	91	61y (range, 35y–87y)	15.1 months (range, 0.8–21.2)	0/91 (0.0%)	N/A	9/91 (9.9%) Hypertension	There is no report of Atrial Fibrillation or Flutter and risk factors are relatively rare in patients other than hypertension and diabetes.	NCT 03206918
An (49)	SA, MC Phase II	R/R WM	44	65y (range, 41y–83y)	33.0 months (range, 2.0–36.5)	0/44 (0.0%)	N/A	8/44 (18.2%) Hypertension	No AF incidence occurred, which indicates its tolerable and manageable therapy safety.	NCT 03332173
Opat (50)	Phase II	R/R MZL	68	70y (range, 37y–95y)	15.7 months (range, 1.6–21.9)	2/68 (2.9%)	1/2 (50.0%) Grade ≥3	2/68 (2.9%) Hypertension	Treatment with Zanubrutinib was associated with fewer dose reductions because of its clinical safety and tolerability.	NCT 03846427
Phillips (10)	SA, MC Phase 1/2	R/R FL MZL	52	MZL 69.5y (range, 52y–85y) FL 63y (range, 38y–79y)	33.8 months	0/52 (0.0%)	N/A	3/52 (5.8%) Hypertension	AEs of AF are unusual in patients with FL or MZL.	NCT 02343120
Song (51)	SA, MC Phase I	CLL/SLL MCL WM FL MZL	44	N/A	31.5 months	0/44 (0.0%)	N/A	1/44 (2.3%) Hypertension	There are no severe side effects like AF or second primary malignancies.	NCT 03189524
Song (52)	SA Phase II	R/R MCL	86	61y (range, 34y–75y)	35.3 months	0/86 (0.0%)	N/A	No Grade ≥ 3 cardiac AEs	There is no evident advance in the incidence rate or severity of AF, and cardiovascular-related AEs are manageable and tolerated.	NCT 03206970

SG, sequential group; MC, multiple-center; SA, single-arm; CLL, chronic lymphocytic leukemia; SLL, small lymphocytic lymphoma; MCL, mantle-cell Lymphoma; FL, Follicular lymphoma; WM, Waldenstrom's macroglobulinemia; MZL, marginal zone lymphoma; R/R, relapsed/refractory disease; N/A, not acquired; AF, artificial fibrillation; AEs, adverse events; BTKi, Bruton's tyrosine kinase inhibitor.

## Discussion

Based on comprehensive analysis results, the critical result of AF incidence is 6.0%. Nevertheless, our findings regarding the incidence rate of AF align with those of other systematic reviews and adhere to similar methodological approaches employed in meta-analyses of bleeding events (53). Meanwhile, through weighted mean, we can also approximately estimate incidence rates of the cardiac-related events: hypertension (14.8%) and heart failure (4.6%). The uncertainty of AEs will affect the prognosis of patients with lymphoma because of the discontinuation of treatment and the high risks for cardiovascular diseases like heart failure and stroke. Previous studies on Ibrutinib have reported common adverse events (AEs) including diarrhea (49%), upper respiratory tract infection (33%), fatigue (32%), cough (31%), and rash (27%) (4). Among these AEs, AF is a type of abnormal heart rhythm that affects the atria. When the atria are not beating coordinatedly, blood may form clots, travel to the brain vessels, and then lead to a stroke (54). Furthermore, it is imperative to underscore the strong correlation between AF and an elevated risk of heart failure, given that irregular atrial contractions can impede the heart's ability to efficiently pump blood. In summation, AF emerges as a salient and pivotal adverse event, particularly within the domain of BTKi treatment for lymphoma for its wide applications and potential risks.

The elevated risks and unique off-target toxicities associated with AF have garnered significant attention, leading to the emergence of a sub-discipline known as Cardio-oncology. This discipline emphasizes the collaboration between hematologists and cardiologists, aiming to enhance the management and prevention of adverse events associated with BTKi. The BTKi-related AF has not been fully discovered. According to certain studies, BTKi-related AF may related to relatively broad selectivity and targets, including BTK, TEC (55), EGFR, and CSK (56). In comparing Ibrutinib and other second-generation BTKi, differences in the target of CSK may lead to the difference in the incidence of AF.

### The first generation BTKi: Ibrutinib

As the original and first-generation BTKi used in the treatment of lymphoma, Ibrutinib has emerged as a successful paradigm and a catalyst for related research. With extensive experimental support and clinical trial data, the reliability of its efficacy in terms of progression-free survival (PFS) and incidence rates of serious adverse events (AEs) has increased. However, notable heterogeneity remains within the Ibrutinib-treated group due to lymphoma types, study designs, and baseline data variations. To conduct further analysis in detail, we categorized the data into more specific subtypes. Nevertheless, the currently available clinical trials have limitations in providing a more nuanced analysis of these subtypes. The reported incidence rate of AF at 10% in Ibrutinib-treated patients can be considered relatively accurate and a reliable clinical reference. Notably, a meta-analysis focusing on AF incidence rate in CLL patients treated with Ibrutinib reported an AF incidence rate of 5% (57). This discrepancy could be attributed to the extensive application of Ibrutinib in lymphoma cases, as CLL was among the earliest approved indications for Ibrutinib, with a relatively low incidence rate of AF. Furthermore, more recent clinical trials have reported rates higher than 10% (25–27). The 10% incidence rate is understandable given the inherent heterogeneity associated with a single-rate analysis.

### The second generation BTKi: zanubrutinib, orelabrutinib, and acalabrutinib

The progress in BTKi therapy has notably lessened treatment complications and bolstered treatment endurance. Zanubrutinib was brought into sharp focus due to its PFS efficiency results or relatively fewer side effects. Safety has become a key factor in the recommendation of lymphoma treatment. Also, based on the NCCN guideline (Version 1.2023) of CLL/SLL, Zanubrutinib is taken as the highest level of clinical recommendation. In contrast, Ibrutinib is taken as another recommendation for its potential cardiovascular risks. According to the result, we may observe that the AF incidence rate is approximately 0% due to the high risks of bias in certain studies. In the head-to-head trials, the AF incidence rate is 2.5% (58). The difference may originate from the limitations of different independent research reports and the methodology of single-group rate analysis. Larger studies, more compact study designs, and real-world data are necessary for further evidence to support the encouraging results of Zanubrutinib.

Orelabrutinib is still at the stage of various clinical trials. Only a few studies disclosed the results, while it is still considered a promising drug as BTKi for more effective and fewer AEs treatment. Although the current results from the single-rate analysis suggest an incidence rate of 0% for AF and no reported cases of AF, it is vital to acknowledge such analyses' limitations and potential biases.

Based on the subtype meta-analysis, the incidence rate of AF with Acalabrutinib in the random effect model is about 4%. Though the induced studies are limited, there is no significant difference between trials or other previous reports. The incidence of AF in the first randomized phase III trial of Ibrutinib (41/263, 15.6%) vs. Acalabrutinib (24/266, 9.0%) may offer us newly updated supportive data.

### New generation BTKi: pirtobrunib

The approval of Pirtobrutinib in January 2023 as a non-covalent BTK inhibitor for MCL patients resistant to covalent ones marks a significant advancement in the third-generation BTKi (59). Also, we may expect the emergence of more third-generation BTKi, including pirtobrutinib (60) and Fenebrutinib (61). These developments highlight the ongoing evolution of BTK inhibitor therapy and the potential for better outcomes in diverse disease contexts. The field of BTKi has evolved from the first generation of exploratory and innovative compounds. Subsequently, the focus shifted towards the second generation of inhibitors, aiming to improve efficacy while minimizing side effects. Presently, the introduction of third-generation non-covalent binding inhibitors represents a significant advancement, expanding the therapeutic indications, and narrowing adverse events.

### Comparative analysis between ibrutinib and zanubrutinib

The US Food and Drug Administration (FDA) and the European Medicines Agency (EMA) warn against the utilization of Ibrutinib for its cardiovascular risks. In the age of targeted therapy, survival time of CLL/SLL patients has been largely prolonged. Thus, monitoring and prevention of cardiovascular events is vital for clinical treatment choices based on comprehensive physical conditions and drug toxicity. Recently, based on a head-to-head experiment (Zanubrutinib vs. Ibrutinib), the incidence rate of AF significantly decreased (2.5% vs. 10.19%, *P* = 0.001). In the study of ALPINE, 6 cases of 652 patients died of cardiac events with Ibrutinib. Also, the PFS in the Zanubrutinib group was better than that in the Ibrutinib group (94.9% vs. 84.0%) (58).

**Figure 2:**
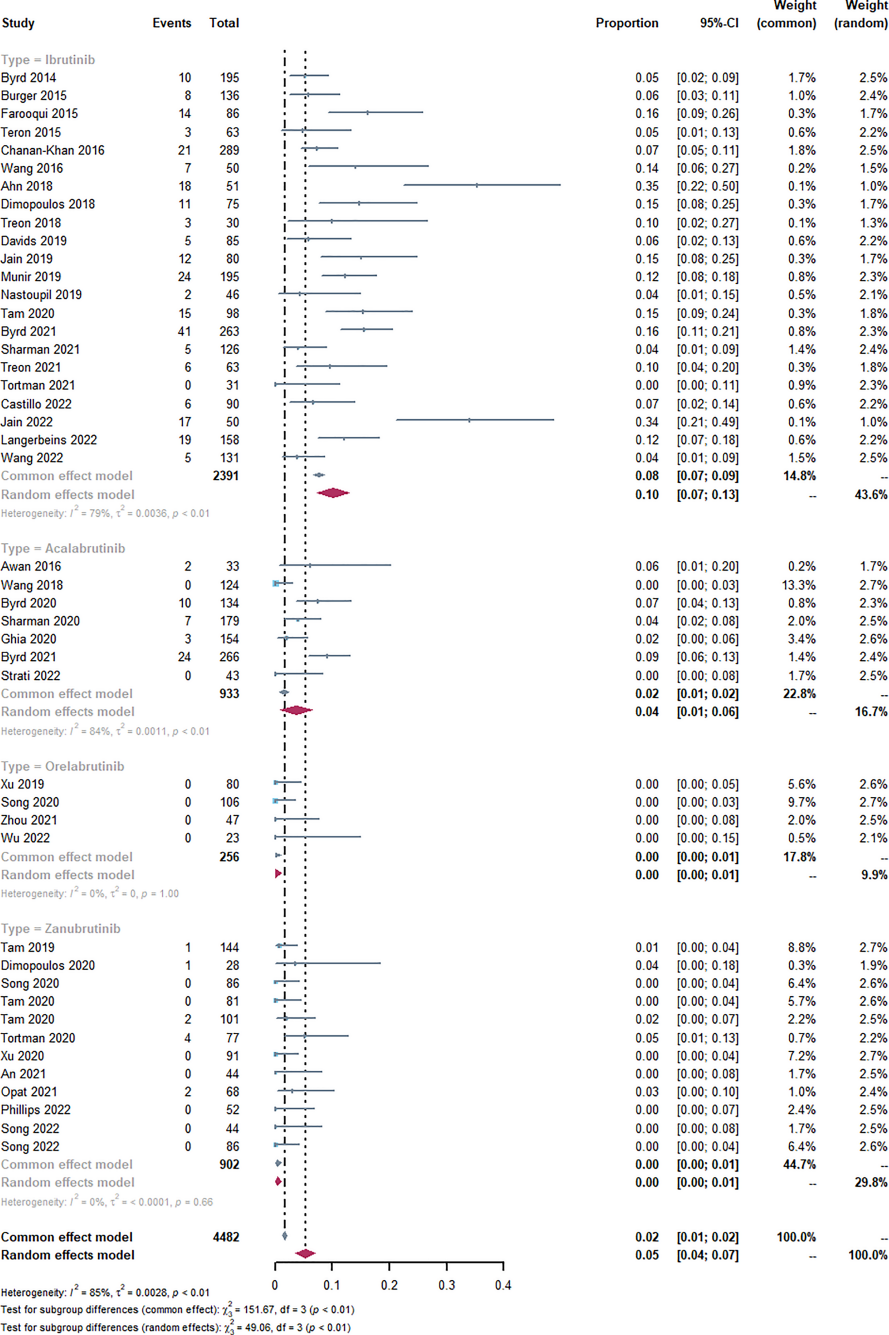
Forest plots of the included studies of atrial fibrillation or flutter occurrence rate and sub-type analysis. Through the single-rate analysis of AF incidence, we determined the AF incidence rates for Ibrutinib (10%, 95% CI 7%–13% in random effects model), Acalabrutinib (4%, 95% CI 1%–6% in random effects model), Orelabrutinib (0%, 95% CI 0%–1% in common effects model), Zanubrutinib (0%, 95% CI 0%–1% in common effects model), and overall AF incidence rate (5%, 95% CI 4%–7% in random effects model).

Through the comparison of Ibrutinib and Zanubrutinib, we may observe a significant difference in that Zanubrutinib performs better in the cardiac safety profiles in Figure 2. Theoretically, we may agree with the higher inhibition effects of Ibrutinib in HER2, HER4, TEC (mainly related to AF) (62) and fewer off-target effects of Zanubrutinib with lower IC50 against BTK (mainly related to the treatment of lymphoma) and higher IC50 for other kinases like EGFR(21nM), TEC(44nM), ITK(50nM) (63). More Head-to-head clinical trials or more detailed disclosure of procedures and results may help us identify their PFS and cardiotoxicity. The different baselines of cardiac risk factors, the ignorant monitoring of AEs, and the median follow-up time may contribute to the difference between the BTKi.

Generally, the incidence rate of AF would increase with longer follow-up time due to its stable long-term risks. However, some studies suggest that AF primarily occurs during a specific period and does not increase over time (62, 64). Comparing the median age and median follow-up time between the two groups, the mean age of both groups is approximately 65.2 months. However, the mean follow-up times differ, with 31.5 months in the Ibrutinib group and 23.38 months in the Zanubrutinib group. Furthermore, future clinical designs and systematic reviews should pay attention to baseline differences and the AF occurrence time.

### Guidance for the management of AF in the treatment of BTKi

The advancements in BTKi have primarily focused on three key areas: developing new generations of BTKi, exploring different dosage regimens, and expanding medication options.

**Figure 3:**
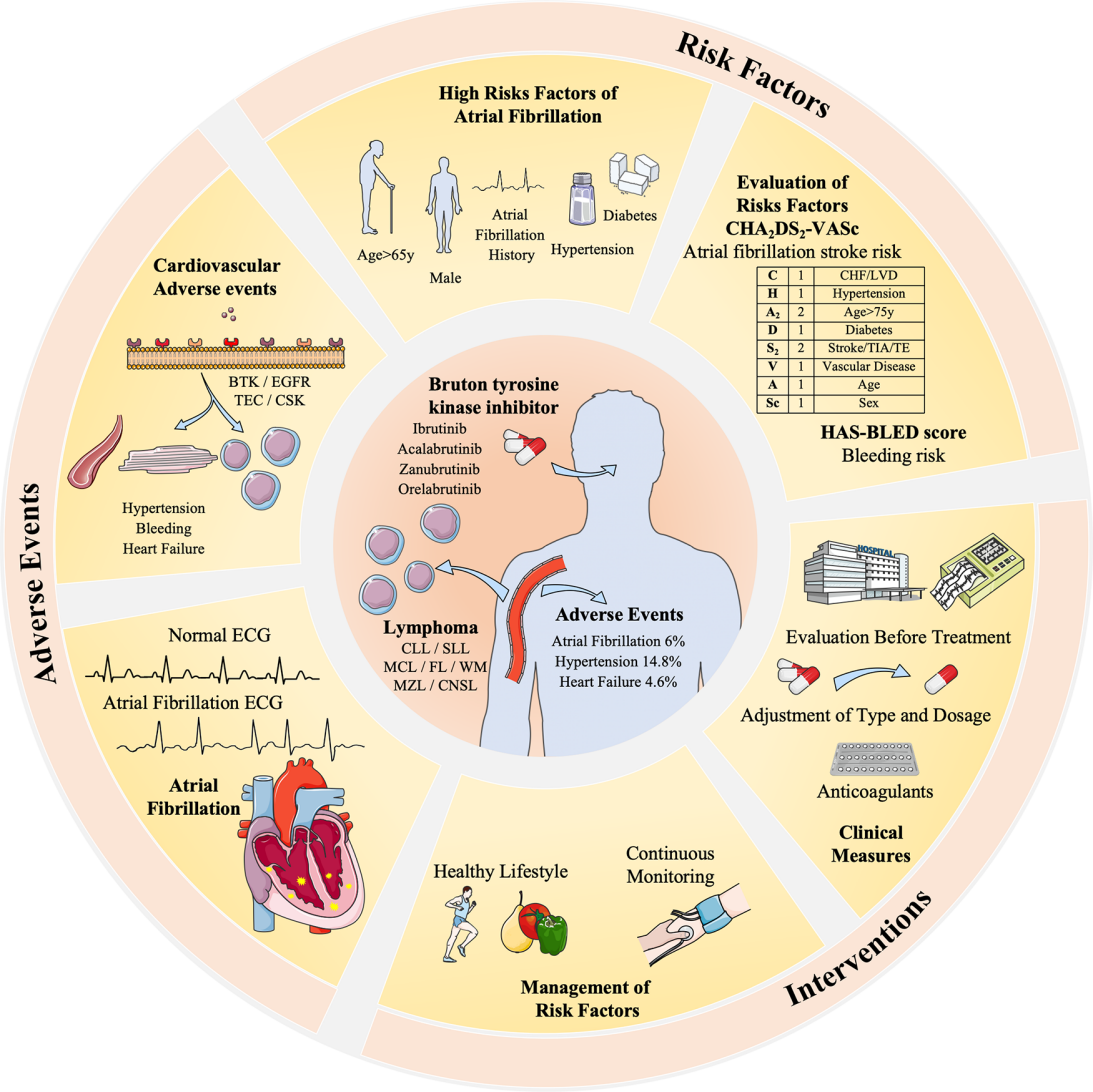
The risk factors, adverse events, and interventions of BTKi-related atrial fibrillation in the treatment of lymphoma. BTK inhibitors are related to different cardiovascular adverse events including AF (5%), hypertension (14.8%), and heart failure (4.6%). It is related to different receptors like BTK, EGFR, and TEC expressed by myocardial cells and vascular endothelial cells. Risk factors and evaluation methods like CHA2DS2-VASc and HAS-BLED are taken to evaluate its interventions. Dosage adjustment or anticoagulants are common clinical measures, and a healthy lifestyle and continuous monitoring are advised to manage risk factors, hypertension, and diabetes. Patients with an AF history are highly risky for BTKi-related AF and require special monitoring and decisions.

However, managing AF as a confirmed AE requires careful consideration before treatment initiation. A thorough assessment of high-risk factors, such as AF history, hypertension, bleeding history, age ≥ 65y, male sex, and underlying diseases, is necessary. Based on a recent study, reported Ibrutinib, age ≥ 65y, blood culture positive, hypertension, diabetes, and sex as risk factors for developed AF in CLL patients (65). Specifically, some studies may focus on AF-risk scores used in patients with CLL, if patients with a score ≥5 should be carefully monitored or changed to second-generation BTKi (66–68). Commonly used clinical tools like the CHA2DS2-VASc and the HAS-BLED aid in evaluating the risk-benefit balance of AF patients in the area of BTKi treatment. For instance, if the CHA2DS2-VASc score exceeds 2, oral anticoagulants such as aspirin are recommended (69). Also, newly developed AF prediction models like HARMS2-AF risk scores can be tested in future trials (70). The personalized treatment plans should be formulated based on patient-specific factors such as scores, electrocardiogram (ECG) results, and blood pressure, including tailored doses and durations of BTKi, anticoagulants, and other supportive treatments (71) (Figure 3).

Moreover, based on clinical needs, early detection methods and specific biomarkers are essential for the management of lymphoma treatment. There are already biomarkers, including Brain Natriuretic Peptide (BNP), ST2, Galectin-3 (72), FGF-23 (73), and microRNAs (74). While we may expect further exploration of the specific biomarker for BTKi-related AF, working as a convenient and inexpensive method. In cases where medically managing BTK inhibitor-related AF proves challenging, discontinuation of BTK inhibitor treatment may be considered. However, this decision must be weighed against its potential impact on treatment efficacy. Thus, striking a balance between side effects and the continuity of treatment is imperative.

## Conclusion

Through a relatively comprehensive collection, we conclude different types of AEs, mainly focusing on the incidence of AF. The incidence rate of AF varies from 0% to 35%, and through a single rate analysis, the overall AF incidence rate is considered as 5% (95% CI 4%-7%). The profiles of BTKi are tolerable and controllable, showing a great advancement in the second-generation BTK with less serious AEs. Also, the cardiac-related AEs indicate us to manage patients regularly and prevent the occurrence of AF during the treatment of lymphoma. In the future, we may further explore the mechanism of BTKi-related AF and find more measures to monitor cardiac-related AEs.
